# Fine-Tuning the Tumour Microenvironment: Current Perspectives on the Mechanisms of Tumour Immunosuppression

**DOI:** 10.3390/cells10010056

**Published:** 2021-01-01

**Authors:** Jesse D. Armitage, Hannah V. Newnes, Alison McDonnell, Anthony Bosco, Jason Waithman

**Affiliations:** 1 Telethon Kids Institute, The University of Western Australia, Nedlands, WA 6009, Australia; jesse.armitage@telethonkids.org.au (J.D.A.); hannah.newnes@telethonkids.org.au (H.V.N.); alison.mcdonnell@telethonkids.org.au (A.M.); 2 National Centre for Asbestos Related Diseases, QEII Medical Centre, The University of Western Australia, Nedlands, WA 6009, Australia

**Keywords:** tumour, immunosuppression, tumour microenvironment, mechanisms, immunotherapy, fine-tuning

## Abstract

Immunotherapy has revolutionised the treatment of cancers by harnessing the power of the immune system to eradicate malignant tissue. However, it is well recognised that some cancers are highly resistant to these therapies, which is in part attributed to the immunosuppressive landscape of the tumour microenvironment (TME). The contexture of the TME is highly heterogeneous and contains a complex architecture of immune, stromal, vascular and tumour cells in addition to acellular components such as the extracellular matrix. While understanding the dynamics of the TME has been instrumental in predicting durable responses to immunotherapy and developing new treatment strategies, recent evidence challenges the fundamental paradigms of how tumours can effectively subvert immunosurveillance. Here, we discuss the various immunosuppressive features of the TME and how fine-tuning these mechanisms, rather than ablating them completely, may result in a more comprehensive and balanced anti-tumour response.

## 1. Introduction

The tumour microenvironment (TME) is a dynamic ecosystem that is manipulated by tumour cells to support its growth and subvert immune surveillance. Tumours exhibit varying degrees of inflammation that can be broadly categorised into immunologically ‘cold’ and ‘hot’ tumours. ‘Cold’ tumours are characterised by increased numbers of immunosuppressive cell types such as regulatory T cells (Tregs), myeloid-derived suppressor cells (MDSCs), M2-switched tumour-associated macrophages (TAMs) and cancer-associated fibroblasts (CAFs), as well as greater extracellular matrix (ECM) density, and poor oxygen and nutrient availability [1]. The ‘cold’ phenotype can be subcategorised into ‘excluded’ tumours, which display limited cytotoxic T lymphocyte (CTL) migration to the tumour periphery but not the tumour core, and ‘ignored’ tumours that are completely devoid of CTLs [2]. Conversely, ‘hot’ tumours present with improved infiltration of CTLs, natural killer (NK) cells, and M1 macrophages as well as increased pro-inflammatory and type-I interferon (IFN-I) signalling [1]. A major focus in the field is to develop new strategies that reprogram the TME toward the ‘hot’ phenotype, which is generally more responsive to immunotherapies including immune checkpoint blockade (ICB) and adoptive cell therapy (ACT). However, treatment-refractory patients commonly exhibit similar or even higher levels of tumour inflammation compared to those that do respond to therapy [3,4], suggesting that there are still significant deficiencies in our understanding of the molecular mechanisms underpinning tumour escape versus control. The TME is intrinsically tuned to resist natural and therapy-induced anti-tumour immune responses. Notably, many of these immunosuppressive mechanisms may also exert anti-tumour effects that are critically overlooked during drug development. This review will specifically highlight the common molecular drivers of the immunosuppressive TME, and the current strategies being investigated to target these pathways. We also discuss the role of these pathways in regulating the balance of inflammatory responses in the TME, highlighting their potential importance in promoting enhanced tumour control.

## 2. Cytokines

### 2.1. Interleukin-10

IL-10 is a homodimeric cytokine that is produced by various cell types including T cells, B cells, macrophages, mast cells, dendritic cells (DCs), granulocytes, and tumour cells [5,6]. IL-10 binds to IL-10 receptor-alpha, activating the Janus kinase (JAK)-Signal transducer and activator of transcription (STAT) and protein kinase B (Akt) signalling cascades, thereby phosphorylating the transcription factor STAT3 which then dimerises and translocates to the nucleus. Additionally, IL-10 signalling mediates the activation of MAFB, a transcription factor that regulates the polarisation of anti-inflammatory macrophages [7]. While recent work has shed light on its immune-stimulating activity [5], IL-10 was initially described as a potent inhibitor of pro-inflammatory cytokine production by activated macrophages and type-I T helper (Th1) cells [8,9]. Effective immune surveillance is governed by the presentation of tumour antigens by DCs to prime CTLs. However, IL-10 is known to interfere with this process by reducing the expression of co-stimulatory molecules or directly dampening T cell receptor (TCR) signalling strength through the re-arrangement of surface N-glycans [10,11,12]. Similar phenomena have also been observed in other antigen-presenting cells (APCs) such as monocytes and macrophages, where IL-10 downregulates the surface expression of CD86 and major histocompatibility complex (MHC)-II proteins. IL-10-treated DCs have been shown to promote CTL anergy toward melanoma-specific antigens, resulting in a loss of cytolytic activity in vitro [11]. Moreover, IL-10-producing monocytes/MDSCs subvert anti-tumour immunity by inhibiting macrophage-derived IL-12 and T cell proliferation in carcinoma models [13,14]. A meta-analysis of over 1700 patients from 21 published studies revealed that elevated serum IL-10 levels were associated with poor prognosis across most solid and haematological cancers [15]. Zhang et al. recently showed that increased IL-10^+^ TAMs in biopsies from gastric cancer patients were associated with poor clinical outcomes and response to chemotherapy [16]. In patients with lung cancer, IL-10 was not only shown to positively correlate with tumour diameter, but it was also demonstrated that IL-10 may counteract intra-tumoural programmed cell death protein 1 (PD-1)/programmed death-ligand 1 (PD-L1) signalling, highlighting the potential role of IL-10 in mediating resistance to immune checkpoint blockade [17]. Despite strong evidence suggesting that IL-10 plays an important role in promoting immune tolerance, numerous studies have also highlighted the anti-tumour potential of IL-10 [18]. This was exemplified by pre-clinical studies showing that IL-10 improves immune surveillance by augmenting the effector functions of intra-tumoural CTLs [19]. Moreover, a recent phase I clinical trial demonstrated that pegylated IL-10 in combination with anti-(α)PD-1 therapy elicited robust anti-tumour immune responses and an improvement in clinical outcomes in patients with renal and non-small-cell carcinoma [20]. Taken together, IL-10 plays a dual role in eliciting immune responses with pro- and anti-tumour properties that need to be precisely regulated.

### 2.2. Transforming Growth Factor-β

The TGF-β cytokine family is composed of three variants (TGF-β1, -β2 and -β3) each of which are initially produced as a latent form before being enzymatically converted to active TGF-β. Each variant interacts with type-I and type-II serine/threonine kinase receptors (TGF-βRI and TGF-βRII), which in turn activates the SMAD pathway. The phosphorylation of SMAD2/3 then facilitates binding to SMAD4 to form the transcriptional complex that accumulates in the nucleus and controls gene expression [21]. TGF-β plays a pivotal role in tumour immunosuppression by impairing the activation and production of cytolytic molecules by NK and CTLs [22,23,24]. Furthermore, TGF-β has been shown to suppress chemokine receptor expression on CTLs, rendering them incapable of trafficking to tumours [25]. TGF-β is also responsible for triggering immunosuppressive cascades by activating forkhead box P3 (Foxp3)^+^ Tregs, MDSCs and CAFs which also have distinct anti-inflammatory properties [26,27,28,29]. Indeed, elevated levels of TGF-β subtypes are associated with poor outcomes across numerous malignancies including cutaneous melanoma, lung, ovarian and triple-negative breast cancer [30,31,32,33]. Due to the major role that TGF-β plays in promoting tumourigenesis, there has been growing interest in therapeutically targeting TGF-β signalling to improve outcomes in cancer patients. Dodagatta-Marri et al. recently showed that resistance to αPD-1 therapy in mice with squamous cell carcinoma was mediated by the induction of immunosuppressive Tregs. However, combinatorial treatment using αPD-1/αTGF-β ablated this effect and was superior in promoting tumour rejection [34]. Similar findings were observed when using dual αPD-L1/LY364947 (a TGF-βRI kinase inhibitor) therapy, although the efficacy of this regimen was found to be more effective against the more immunogenic MC38 colon adenocarcinoma cell line, highlighting a further limitation of the strategy [35]. This synergistic effect with ICB has also led to the development of bifunctional agents that simultaneously block PD-L1 and TGF-βRII which has shown promising results in pre-clinical models and phase I clinical trials [36]. Although TGF-β blockade has shown promising results in combination with other immunotherapeutic strategies and in pre-clinical models, these findings have yet to be fully recapitulated across numerous clinical trials [21]. One possible explanation for this is the direct effect that TGF-β blockade has on cancer cells, given that the loss of TGF-β signalling promotes mesenchymal–epithelial transition—an important step during metastasis [37]. Secondly, TGF-β can be shuttled via extracellular vesicles, rendering them inaccessible to antibody-based therapies [38]. Lastly, TGF-β plays a crucial role in the formation and maintenance of CD8^+^ tissue resident memory T cells (T_RM_) that are a critical component of the anti-tumour response [39]. While TGF-β has been classically defined as an anti-inflammatory agent, contrary findings have shed light on its role in tempering tumour outgrowth and promoting immune homeostasis within the TME.

### 2.3. Type-II Inflammatory Cytokines

Type-II inflammation plays a critical role in modulating the anti-tumour immune response. An imbalance toward type-II inflammation has been associated with the ‘cold’ tumour phenotype compared to ‘hot’ tumours that are enriched with the more cytotoxic type-I inflammation across multiple cancers [40]. Alarmins such as thymic stromal lymphopoietin (TSLP) and IL-33 are essential upstream regulators of the type-II inflammatory response that are produced by stromal and endothelial cells in response to stress and cellular damage. TSLP signals through TSLP-receptor (TSLPR) that is found on group 2 innate lymphoid cells (ILC2s), T cells and APCs [41]. Similarly, IL-33 is a member of the IL-1 family and binds to the ST2 receptor found on ILC2s and T cells [42]. Tumour cell-derived TSLP reprograms myeloid DCs with Th2-polarising activity, and treatment of these mice with αTSLP antibodies not only diminished this effect but also significantly prolonged survival in humanised mice [43]. TSLP receptor (TSLPR)^o/o^ mice engrafted with 4T1 breast tumour cells restored type-I immune responses resulting in slower 4T1 tumour growth and reduced lung metastasis [44]. Takahasi et al. also demonstrated that fibroblast-derived periostin could stimulate TSLP by keratinocytes and promote a Th2-dominant TME in a model for cutaneous T cell lymphoma [45]. Production of TSLP by CAFs within the TME and IL-4 by basophils in draining lymph nodes (dLNs) drives the polarisation of Th2 CD4^+^ T cells and M2 macrophages [46,47]. The IL-33/ST2 axis on Tregs is a key tumourigenic driver in both skin and colon cancer [48,49], while ST2 deletion enhanced type-I inflammatory responses that promote clearance of murine mammary carcinoma [50]. This pathway is also responsible for the polarisation of matrix metallopeptidase 9-secreting M2 macrophages which accelerate adenocarcinoma metastasis [51]. While the expression of alarmins in the TME have been classically associated with increased tumour growth and metastasis, contrary evidence has also positioned them as important regulators of anti-tumour immunity. TSLP elicits protection against skin carcinogenesis by enhancing dermal T memory cell immunity [52], which may not be surprising considering TSLPR naturally complexes with the IL7R to promote CTL memory formation and homeostasis [53,54]. A similar skin cancer model also demonstrates that TSLP enables Th2 formation which exerts an anti-tumour effect and improves tumour surveillance [55]. In an elegant model published by Demehri et al., transgenic K14 mice overexpressing dermal TSLP could successfully arrest breast and pancreatic tumour development, which was associated with an influx of GATA3^+^ Th2 cells to the primary tumour site [56]. Similarly, tumour growth was markedly reduced following IL-33 treatment, which was attributed to an increase in the migration and viability of cytotoxic eosinophils in a model for colorectal cancer [57]. In addition, IL-33 has been shown to drive anti-tumour CTL and NK cell activity that reduces melanoma tumour growth and metastasis [58]. Xia et al. further demonstrated that exogenous IL-33 recapitulates CTL effector functions in IL-33-deficient mice in a colon carcinoma model [59]. Further reports indicate that ST2 expression on CTLs is promptly upregulated following polarisation by type-I inflammation, while IL-33 and IL-12 (a canonical type-I inflammatory cytokine) act synergistically to augment the effector functions of CTLs [60]. This critical finding suggests that a balance of type-I and type-II inflammation is essential for optimal immune function. Canonical type-II inflammatory cytokines, including IL-4, IL-5, IL-13 are primarily produced by Th2 cells and ILC2s. Type-II inflammatory cytokines, including alarmins in the TME is tightly linked with the induction of M2 macrophages and Tregs [61,62]. Additionally, ILC2 numbers have been shown to correlate with infiltrating MDSCs in recurrent bladder cancer, where ILC2-derived IL-13 upregulated the expression of T cell-suppressing genes including ARG1 (encoding arginase-I; ARG1) and NOS2 (encoding inducible nitric oxide synthase; iNOS) [63]. Durable responses to ICB correlate with improved Th1/Th17 activity and a concomitant suppression of Th2 immunity [64]. One novel approach to circumvent the type-II-skewed TME is to utilise Inverted Cytokine Receptor (ICR)-modified CAR T cells that arm, rather than attenuate the cancer-killing mechanisms of T cells in the presence of Th2 cytokines. Using this strategy, IL-4/21 ICR (where the ectodomain of the IL-4R is fused to the endodomain of the IL-21R) CAR T cells were significantly more effective at eradicating IL-4^+^ tumours in vivo [65]. It is noteworthy that type-II inflammatory cytokines may be beneficial in eliciting anti-tumour immune responses. For instance, recent work has demonstrated that targeted knockdown of TGF-βRII expression on CD4^+^ T cells augments anti-tumour responses and vascular remodelling in an IL-4-dependent manner [66,67]. A recent study by Moral et al. supports this further by showing the ILC2s in pancreatic ductal adenocarcinoma (PDAC) cells are highly receptive to PD-1 blockade and augment tissue-specific tumour immunity [68]. Remarkably, adoptive transfer of tumour-reactive Th2 cells were successful in promoting tumour rejection [69]. This is corroborated by other work demonstrating that memory Th2 cells potently activate cytotoxic NK cells to slow tumour growth [70], suggesting that a complete ablation of type-II inflammation within the TME may be detrimental to immune surveillance.

## 3. Metabolites

### 3.1. Tryptophan and Kynurenine

Catabolism of the essential amino acid tryptophan (Trp) by indoleamine 2,3-dioxygenase-1 (IDO1) is the first and rate-limiting step in the synthesis of nicotinamide adenine dinucleotide (NAD) which is a critical co-factor involved in glycolysis and oxidative phosphorylation [71]. Immune suppression is triggered by a two-fold effect of IDO activity by first depleting Trp, and the accumulation of the immunosuppressive metabolite kynurenine (Kyn). IDO1 is an inducible enzyme produced by a broad range of myeloid cells (including DCs and macrophages), endothelial cells, mesenchymal stromal cells and fibroblasts [72]. Increased IDO1 has been shown to stifle the activation of T cells, inhibit NK cell function, stimulate Tregs, induce tolerogenic DCs, expand MDSCs and neovascularisation [73]. The Trp starvation theory proposes that depletion of Trp facilitates accumulation of uncharged tRNA and activation of the general control nonderepressible 2 (GCN2) pathway and subsequent T cell dysfunction [74]. Trp depletion has been shown to inhibit mammalian target of rapamycin (mTOR) and protein kinase C in cancer cells and enhance autophagy and Treg development [75]. Furthermore, a loss of mTOR signalling may shift the balance of the CTL compartment toward short-lived and suboptimal cytolytic responses [76]. Accumulation of Kyn is important in maintaining peripheral homeostasis and tempering inflammatory lymphocyte activity. The Aryl hydrocarbon receptor (AhR) pathway is activated by Kyn which is toxic to lymphocytes and induces CD4^+^ T cell differentiation into Tregs [72,77]. AhR dimerises with the AhR nuclear translocator (ARNT) protein to bind multiple transcriptional co-factors to drive transcription of IL-10 in DCs and NK cells and IL-6 in macrophages and cancer cells [78,79]. As previously described, IL-10 has a dichotomous role in the TME by eliciting both pro- and anti-tumour responses. Interestingly, tumour cells expressing high levels of IDO were observed to have a slower growth rate compared to low IDO expressing cells [80]. Furthermore, higher intra-tumoural expression of IDO has been correlated with longer survival in multiple cancer types [81,82,83] but also worse prognosis in several other cancers [84,85,86,87,88]. The effect of IDO as an immunosuppressive, pro-tumour factor has been queried due to a lack of efficacy reported in a recent phase III clinical trial combining the potent IDO inhibitor epacadostat with αPD-1 therapy in patients with non-resectable and metastatic melanoma [89].

### 3.2. Adenosine

Purinergic signalling is tightly regulated by the activity of surface ectonucleotidases, CD39 and CD73. The pair of enzymes are responsible for the conversion of extracellular adenosine triphosphate (eATP) to adenosine. The release of eATP is a common consequence of cellular stress including inflammation, hypoxia or ischemia which triggers adenosine accumulation. Adenosine elicits immunosuppression predominantly through the type-I purinergic receptors, A2_A_ and A2_B_ as part of a regulatory negative feedback loop [90]. Indeed, the accumulation of adenosine and the expression of CD39 and CD73 are well described features of the TME [91]. A broad range of immune cells express adenosine receptors including T cells, NK cells, natural killer T cells (NKTs), macrophages, DCs, neutrophils, mast cells and B cells [92]. The A2_A_ receptor has been shown to promote proliferation and immunosuppressive function of Tregs [93,94], inhibit T cell proliferation, cytotoxicity and inflammatory cytokine production [95]. The CD39/CD73 axis fine-tunes macrophage differentiation and activity by promoting M2 polarisation [96], while A2_B_ agonists promoted infiltration of MDSC in melanoma-bearing mice which was diminished following A2_B_ blockade [97]. Furthermore, CD73-deficient mice are resistant to carcinogenesis, while metastasis of CD73^+^ tumours is significantly impaired following A2_A_ blockade [98,99]. However, in contrast to the established role of adenosine in generating an immunosuppressive TME, other work has highlighted its importance in T cell differentiation. Deletion of A2_A_ receptors increased tumour growth and impaired CTL and differentiation in a B16F10 tumour model [100]. This is supported in other studies which demonstrate that adenosine signalling favours the generation of long-lived memory T cell precursors that are protected against ATP-induced apoptosis [101,102]. Increased expression of CD73 has been observed in a wide range of cancers and correlated with a worse prognosis [103]. However, contradictory to such evidence, Ineoue et al. [104] found that tumour CD73 and A2_A_ protein expression in lung adenocarcinoma associated with a better prognosis. Further confusing the prognostic value of CD73, studies have shown expression correlated with better prognosis in ovarian and breast cancer [105,106]. Nevertheless, the dual-blockade of CD39 and CD73, in combination with αPD-1/αCytotoxic T lymphocyte-associated protein-4 (CTLA-4) was successful in promoting robust anti-tumour T cell immunity in known therapy-resistant cancer models [107]. The authors also noted that the accumulation of eATP following CD39/CD73 blockade promoted the activation and maturation of DCs and M1 macrophages [107]. This has also led to the development of clinical trials targeting the A2_A_ receptor in patients with refractory renal cell cancer [108]. In summary, blocking adenosine signalling is a feasible strategy that favourably re-shapes the immune landscape of the TME, although contradictory findings do suggest that the preservation of this pathway might be important in regulating T cell homeostasis and the maintenance of long-lived memory T cell precursors.

### 3.3. Nitric Oxide

Inducible nitric oxide synthase (iNOS) is a key enzyme involved in the production of nitric oxide (NO) and is expressed by an array of cells including macrophages, MDSCs, DCs, NK cells, tumour cells and endothelial cells [109]. NO is critical in many physiological functions but also has been shown to drive a dual role in tumour development. iNOS is promptly upregulated upon exposure to external stimuli (such as lipopolysaccharide; LPS), hypoxia and proinflammatory cytokines (IL-1, IFN-gamma; IFNγ), tumour necrosis factor-alpha; TNFα) resulting in the production of large quantities of NO [110]. Over-expression of iNOS has been associated with poor prognosis in a series of human cancers [111,112,113]. Furthermore, elevated iNOS gene expression in patients has been demonstrated in numerous cancer types, which was contrasted by a lower expression in surrounding healthy tissue [114,115]. However, other attempts to correlate iNOS expression with patient outcomes has led to contradictory results, and additional studies have questioned its value as a prognostic marker [116,117]. Work by various groups have clearly demonstrated the suppressive effects of NO on T cell function via different mechanisms including the inhibition of the critical JAK3/STAT5 signalling pathway, inhibition of MHC class II expression and induction of T cell apoptosis [118,119,120]. Moreover, NO crucially recruits MDSCs, Tregs, M2 macrophages and Th2 cells to the TME to propagate the ‘cold’ tumour niche [121]. Increased expression of NO mediates the upregulation of vascular endothelial growth factor (VEGF) signalling in the TME which promotes tumour growth and invasiveness [110,122]. Xiong et al. demonstrated that NO also inhibited the production of IL-12 in DCs and M1 macrophages [123]. Multiple groups have demonstrated increased type-I immunity and IL-12 production in iNOS knockout mouse models after bacterial infection. Intriguing work from Marigo et al. demonstrated the local iNOS-expressing DCs cooperate with adoptively-transferred CTLs to orchestrate tumour killing. This study also demonstrated that the expression of the iNOS-encoding gene, NOS2 correlated with improved T cell density in tumours and disease-free survival in patients with colorectal cancer [124]. Klug et al. indicated the ability of low dose irradiation in vivo to polarise iNOS^+^ M1 macrophages, which promoted type-I immunity and improved CTL infiltration via NO-dependent vascular remodelling [125]. Notably, these findings challenge other lines of evidence showing that the presence of reactive nitrogen species in tumours, such as NO can impair CTL infiltration via the nitration of T cell-attracting chemokines [126]. Expression of iNOS in CD4^+^ T cells has also been reported to suppress Treg accumulation in pre-clinical cancer models and disrupt tumour tolerance by inhibiting production of TGF-β1 [127]. NO has been classically recognised as a myeloid-derived immunosuppressive molecule that inhibits T cell survival, function, and migration. However, conflicting reports also show that NO is indispensable to anti-tumour immunity which is likely determined by its precise concentration and spatiotemporal abundance in the TME.

### 3.4. L-Arginine

Arginase-1/2 (ARG1/2) promotes the catabolism of the amino acid arginine (L-Arg) into urea and ornithine, which is subsequently broken down into proline and polyamines to drive collagen synthesis and cell proliferation, respectively [128]. Regulatory myeloid cells such as M2 macrophages and MDSCs are recognised as the primary regulators of L-Arg metabolism through the expression of ARG1 during infection and inflammation [129]. Expression of ARG1 is promptly upregulated in these cells in response to Th2 and anti-inflammatory cytokines (including IL-4, IL-13, IL-10 and TGFβ) to assist in resolution of inflammation and promote tissue repair [130]. Many studies have in fact correlated overexpression of ARG1/2 and poor prognosis in a variety of cancers types [131,132,133,134,135,136]. Notably, the deprivation of L-Arg has been shown to have a direct detrimental effect on tumour growth by promoting autophagy, apoptosis and cell cycle arrest [137]. However, it is also well described that the loss of L-Arg metabolism has a profound effect on anti-tumour immunity. Deprivation of L-Arg from the microenvironment by regulatory myeloid cells has a profound effect on the local immune landscape. ARG1 expression by MDSCs favours the generation of IDO-expressing, tolerogenic DCs [138], while L-Arg deficiency compromises CD3 zeta chain (CD3ζ) expression on T cells, subsequently impairing TCR signalling, proliferation and IFNγ production [139]. Furthermore, L-Arg availability shifts the metabolic programs of T cells toward oxidative phosphorylation to promote the generation of a central memory phenotype that are endowed with improved survival and anti-tumour activity in a B16 melanoma model [140]. Indeed, the inhibition of ARG1/2 activity has yielded positive results across numerous cancer models by reducing myeloid-driven immune suppression [134,141]. However, adoptive transfer of Arg2^−/−^ CTLs was also more efficient at clearing tumours and synergised with PD-1 blockade, suggesting that CTL-intrinsic ARG2 activity contributes to the suppression of their anti-tumour activity [142]. To date, phase I clinical trials testing arginase inhibitors have shown some promise in boosting anti-tumour immunity. However, its progress to the clinic has been limited partly due to the essential role of ARG in metabolising ammonia which is highly toxic [143,144].

### 3.5. Prostaglandin-E2

**Figure 1:**
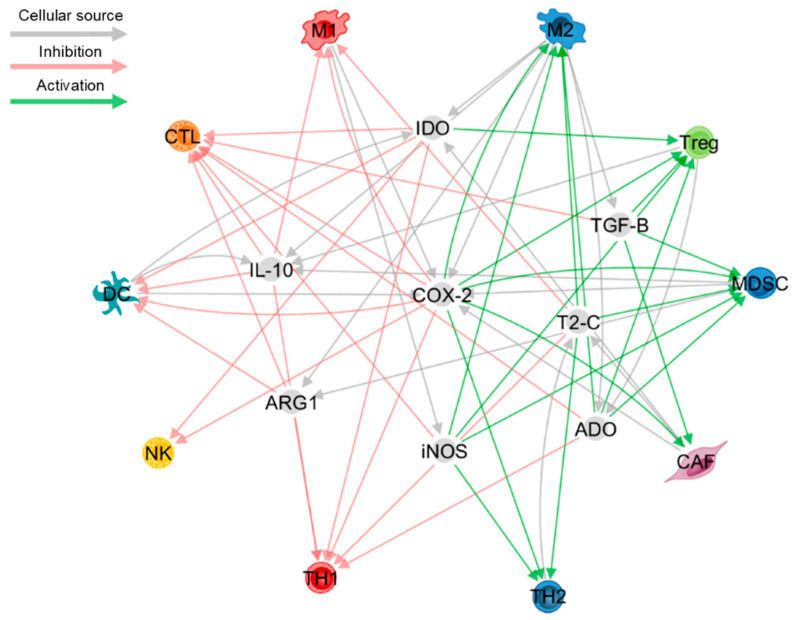
The immune circuitry within ‘cold’ tumour niches is governed by soluble factors such as cytokines, growth factors and enzyme-regulated metabolites. Immune and stromal cell types send and receive signals within the tumour milieu which culminates in the generation of an immune privileged TME that impairs anti-tumour immunity. Abbreviations: T2-C: type-II inflammatory cytokines, IDO: indoleamine 2,3-dioxygenase, TGF-B: transforming growth factor β, ADO: adenosine, ARG1: arginase-1, iNOS: inducible nitric oxide synthase, and IL-10: interleukin-10.

Prostaglandin-E2 (PGE_2_) is a bioactive lipid generated by cycloxygenase-2 (COX-2) following the enzymatic conversion from arachidonic acid. In the TME, PGE_2_ is predominantly synthesised by myeloid, stromal and cancer cells, and signals through the G protein-coupled receptor group, EP1-EP4 [145]. PGE_2_ is recognised as a pan-immunosuppressive mediator as it inhibits CTLs, NK cells and type-I inflammation, while promoting Treg, MDSC expansion and type-II inflammation [146]. Indeed, pilot data from a phase I clinical trial has shown that small-molecule inhibitors of EP4 are well tolerated and slowed disease progression in a proportion of patients with advanced cancers [147], while pre-clinical studies have demonstrated that COX-2 inhibition synergises with ICB to improve tumour eradication, highlighting its potential as a therapeutic adjuvant [148]. Despite strong evidence for its role in dampening anti-tumour inflammation, PGE_2_ has been implicated in regulating the memory T cell compartment which is an important axis of tumour control. For instance, PGE_2_ treatment of umbilical cord blood (UCB) cells lead to the expansion of TCF7- and eomesodermin (EOMES)-expressing CTLs that display a stem-like phenotype, thereby improving the immune reconstitution efficiency of UCB transplantation [149]. In contrast to what has been previously reported, PGE_2_ selectively inhibits the expansion of certain Treg subtypes, revealing a pro-inflammatory role for PGE_2_ [150,151]. Furthermore, PGE_2_ has been shown to restore CCR7-dependent migration of DCs to dLNs, which subsequently improves CTL-DC crosstalk in a prostate cancer model [152]. This is complicated further by more recent data demonstrating that TLR agonists in combination with PGE_2_ promotes mature, cytokine producing DCs with impaired antigen cross-presentation activity [153]. These divergent findings may suggest that nominal PGE_2_ signalling, possibly through certain EP receptors may be beneficial in certain aspects of anti-tumour immunity. Collectively, PGE_2_, in combination with other soluble factors play a fundamental role in creating an immunosuppressive TME (Figure 1).

## 4. Impairment of IFN-I Signalling

Type-I interferons (IFN-Is) are a functionally diverse family of cytokines that play a crucial role in generating potent innate and adaptive immune responses against cancer [154]. IFN-Is are indispensable to anti-tumour immunity by enhancing intra-tumoural CTL-DC crosstalk [155], as well as the augmentation of NK and M1 macrophage activity in the TME [156,157]. ‘Cold’ tumours are generally characterised by poor immune cell infiltration and an accumulation of immunosuppressive factors within the TME. Consistent with this theme, ‘cold’ tumours also restrict endogenous IFN-I activity. In biopsies from patients with triple-negative breast cancer, tumour-associated plasmacytoid DCs (pDCs) produced significantly lower amounts of IFNα, which correlated with the accumulation of intra-tumoural Tregs [158]. IFN-I production by pDCs has also been shown to be compromised in the presence of TGF-β and TNFα within breast tumours [159]. Katlinski et al. demonstrated that the hypoxia-induced downregulation of IFN alpha receptor-1 (IFNAR1) in the TME was a central mechanism that impedes the viability of CTLs, generating ‘cold’ tumour niches [160]. Higher expression of interferon regulatory factor-7 (IRF7) gene signatures in primary tumours has also been linked to prolonged bone metastasis-free survival in breast cancer [161]. Indeed, the efficacy of current cancer therapies such as radiotherapy, chemotherapy and immunotherapy rely on intact IFN-I signalling within tumours [162,163,164]. Accordingly, agents that induce IFN-I responses (such as poly-I:C and stimulator of IFN genes; STING agonists) are used widely as adjuvants for current therapies with moderate success [165,166]. Despite this, there has been mounting evidence that IFN-I signalling also exerts a detrimental effect on anti-tumour immunity. Persistent tumour IFN signalling has been shown to drive adaptive resistance to ICB, which was diminished when mice were pre-treated with JAK inhibitors to block downstream IFN signalling [167]. Furthermore, inflammatory breast cancer (which is a rare and aggressive form of breast cancer) is frequently associated with hyper-activation of IFN-I pathways [168]. More recently, Effern et al. noted that recurring tumours with significant loss of antigen expression were associated with more intense IFN signalling [169], suggesting that tumour dedifferentiation as a mechanism of immune evasion may be driven by overexuberant anti-tumour responses within the TME. This may also be confounded by the functional heterogeneity of IFN-Is, where specific IFNα subtypes are clearly more potent primers of the anti-tumour immune response, although this has only been explored in murine models [154].

## 5. Hypoxia

The establishment of a hypoxic TME is a hallmark of solid cancer progression and dramatically re-shapes the immune contexture of the TME. The cellular response to hypoxia is driven largely by hypoxia-inducible factors (HIF-1α, -2α, -3α) which are oxygen-sensitive transcription factors that are stabilised in the presence of low oxygen concentrations [170]. The relationship between hypoxia and immune suppression in the TME is well established and is strongly linked to numerous mechanisms described above, including the impairment of IFN-I signalling, upregulation of immune checkpoint molecules and the upregulation of extracellular adenosine [170]. Elevated hypoxic gene signatures are a major prognostic indicator in cancer patients and are frequently associated with immune-privileged (‘cold’) tumour niches [171,172]. Hypoxia and the induction of lactate metabolism has been reported to promote M2 polarisation of TAMs via the activation of HIF-1, Hedgehog and mTOR pathways [173,174]. In addition, tumour cell-derived exosomes under hypoxic conditions were also able to reprogram macrophages toward an M2 phenotype [175]. While it has been demonstrated that elevated HIF-1α signalling in DCs results in Th2-biased activation, decreased antigen uptake, and decreased CTL expansion [176,177,178], other work has shown that hypoxic stress improved dLN trafficking of DCs via CCR7 and promoted a highly pro-inflammatory gene expression profile [179,180]. These divergent findings may be at least partially explained by the activation of HIF-1α-independent pathways and the prevalence of other metabolic confounders in the TME that subvert cellular function (i.e., the depletion of glucose, low pH, etc.). Of note, HIF-1α also acts a negative regulator for pDC development, limiting IFNα production [181]. Hypoxic zones within the TME are significantly more resistant to CTL penetrance, which represents a major challenge in T cell-based immunotherapies [182]. The exclusion of CTLs from these regions rely heavily on the accumulation of immunosuppressive myeloid cells and Tregs that are dependent on various cytokines such as CCL28, CCL5 and TGF-β [183,184,185]. Knockdown of hypoxia-induced triggering receptors expressed on myeloid cells (TREM)^+^PD-L1^+^ TAMs in advanced hepatocellular carcinoma (HCC) could successfully rescue CTLs from an exhaustive state and restore their cytolytic activity, which was largely attributed to a reduced recruitment of Tregs to the TME [186]. This is supported by other studies showing that the use of oxygen supplementation or hypoxia-disrupting drugs in combination with ICB can also successfully mitigate this effect by reducing MDSC density [182,187]. Hypoxic stress, mediated by HIF signalling has also been shown to directly regulate T cell differentiation by favouring glycolytic metabolism and effector transition that eventually leads to exhaustion [188]. CART cells, under hypoxic conditions in vitro displayed significantly less proliferative and cytokine-producing activity compared to those under normoxia [189]. Moreover, hypoxia has been shown to induce defects in mitochondrial function that lead to CTL exhaustion [190]. Conversely, Palazon et al. demonstrated that intrinsic HIF-1α signalling was essential for CTL infiltration and effector function, highlighting the importance of HIF-1α in the adaptation of CTLs to the hypoxic TME [191]. While this seemingly contradicts the detrimental role of hypoxia and HIF signalling in CTLs, it is important to consider that tumour cells can out-manoeuvre this metabolic adaptation by also depleting glucose from the TME, thereby diminishing the activity of CTLs that rely heavily on glycolysis for energy production [192]. Interestingly, CTLs reprogrammed to utilise fatty acid catabolism under oxygen and glucose deprivation could recapitulate their cytolytic activity, rendering them more responsive to ICB [193]. These metabolic changes in the TME also have a direct effect on tumour cells and their ability to evade immune responses, as combined oxygen and glucose starvation decreases their presentation of tumour antigens on MHC class I to avoid recognition by CTLs [194]. Hypoxia and various mechanisms of immune suppression interface heavily and mutually support each other to promote tumour growth, highlighting the enormous challenge presented when designing new therapies that successfully address both issues.

## 6. Extracellular Vesicles

Extracellular vesicles (EVs) are lipid bilayered nanoparticles that shuttle various bioactive components, such as RNAs, proteins and lipids between cells. Tumour-derived EVs (T-EVs) bearing Fas ligand (FasL) and TNF-related apoptosis-inducing ligand promote CTL apoptosis [195,196,197]. Similarly, T-EVs expressing membrane-associated TGF-β1 enhanced Treg function while impairing NK and CTL activation [198]. Other lines of evidence demonstrate that T-EVs promote M2 macrophage polarisation which accelerates cancer growth and metastasis [199,200]. Moreover, exosomes produced by these macrophages have been shown to contain micro-RNAs (miRNAs) that dysregulate the balance of Treg:Th17 cells in ovarian cancer [201]. EVs produced by other anti-inflammatory cells such as Tregs and MDSCs also dampen CTL and type-I inflammatory responses by delivering various miRNAs or immunomodulatory proteins [202,203,204], suggesting that EVs produced by both tumour and immune cells in the TME help propagate the ‘cold’ tumour niche to suppress anti-tumour immunity. EVs within the TME are also major mediators of therapy resistance in cancer patients. Richards et al. showed that CAFs-derived EVs could elicit chemotherapy resistance in PDAC cells via the upregulation of the Snail pathway [205], while similar resistance to chemotherapy has been demonstrated via the delivery of macrophage-derived EVs containing miR-21 and miR-365 [206,207]. In a melanoma model, tumour cells shedding EVs mediated chemotherapy resistance by promoting M2 macrophage polarisation and upregulating *IL10* and *ARG1* expression in stromal cells [208]. Additionally, PD-L1^+^ EVs produced by cancer cells are a prominent mechanism of immunotherapy resistance by acting as off-target decoys for αPD-1 monoclonal antibodies that are used to reinvigorate anti-tumour T cell immunity [209]. Although targeting EVs within the TME may be a plausible approach to ameliorating tumour immunosuppression, a critical study by Wolfers et al. showed that T-EVs deliver tumour antigens to DCs to enable CTL cross-priming [210]. Other work has highlighted the role of DC-derived EVs in delivering peptide-loaded MHC and co-stimulatory molecules to cancer cells, improving their immunogenicity [211].

## 7. Exclusion of T Cells from the Tumour Bed and Disruption of T Cell Homeostasis

**Figure 2:**
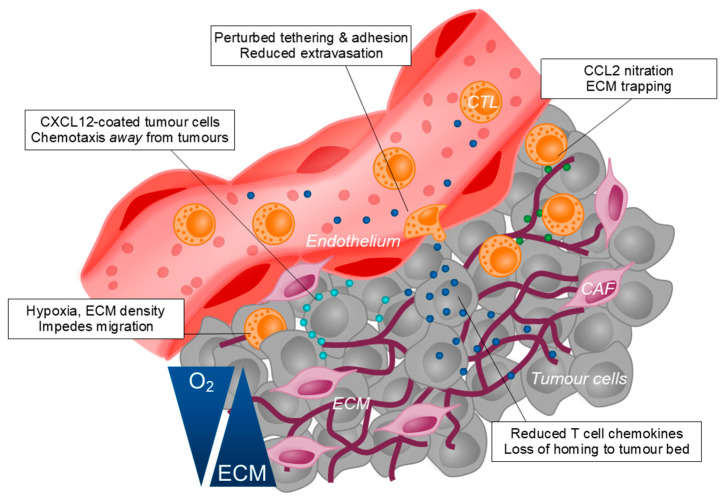
The TME perturbs multiple mechanisms of T cell migration to avoid immune surveillance. Cancer cells can impede with CTL trafficking to the tumour bed at multiple levels including the loss of extravasation capacity, disrupted chemokine gradients and physical constraints including increased ECM deposition and poor oxygen availability.

T cells, in particular CTLs are considered one of the key effectors in mediating anti-tumour immunity. Indeed, a defining characteristic of ‘cold’ tumours is the exclusion of CTLs from the tumour bed. Several factors contribute to the impairment of CTL infiltration into tumours, including mechanisms described above such as hypoxia and the accumulation of anti-inflammatory cells. These conditions that are hostile to CTLs also disrupt the chemokine signalling pathways that are essential to CTL trafficking. TME-residing MDSCs promote the nitration of CCL2 via the production of reactive nitrogen species, impairing the trafficking of CTLs to the tumour site and trapping them in the surrounding stroma [126]. Additionally, increased concentrations of CCL27, CCL5 and CXCL10 in tumours have been associated with better mobilisation of CTLs to the TME [212,213,214]. Tumour cell-derived galectins have been shown to impair the activities of IFNγ-induced chemokines, CXCL9/10/11 by decorating ECM glycans and subsequently trapping intra-tumoural IFNγ [215]. Conversely, CAFs can directly impede CTL trafficking by secreting CXCL12 which, at high concentrations deters CTL migration [216]. The tumour vasculature also undergoes significant remodelling to stifle the migration of CTLs to the tumour bed. Upregulation of VEGF, IL-10 and PGE_2_ at the tumour site cooperatively promotes Fas ligand expression on tumour endothelial to elicit apoptosis of CTLs, but not Tregs [217]. Furthermore, VEGF signalling and local NO production induces defects in the structural arrangement of adhesion molecules on tumour endothelial cells to impair CTL extravasation [218]. Lastly, the ECM architecture laid out by CAFs physically constrains CTLs to areas of lower collagen and fibronectin density, which was reversed following collagenase treatment [219]. Collectively, tumour cells can manipulate its local milieu to suppress multiple mechanisms of CTL migration to the TME (Figure 2).

**Figure 3:**
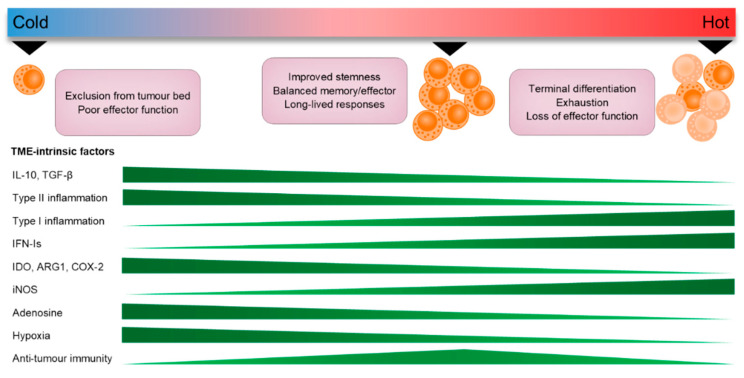
Tumours with intense inflammation may perturb the homeostatic balance of memory-effector T cell populations in the TME. ‘Cold’ tumour niches contain an abundance of canonical immunosuppressive factors that create an immune-privileged TME. Conversely, ‘hot’ tumours that contain excessive amounts of pro-inflammatory factors may disrupt the balance of effector-memory CTL populations, resulting in short-lived effector responses. In contrast, a balance of pro- and anti-inflammatory signals in the TME may endow CTLs with improved cytolytic responses that are long lived.

CTLs that do successfully migrate into the TME are required to integrate an array of pro- and anti-inflammatory signals to appropriately endow them with cancer-killing activity. Infiltrating CTLs that maintain memory and stem-like properties are superior in mediating long-term anti-tumour immunity, while durable response to immunotherapy relies on the expansion of these subsets [220,221]. While it is indisputable that ‘hot’ tumours are skewed toward a Th1, pro-inflammatory phenotype that supports the effector functions of CTLs, there is emerging evidence that many “pro-tumour” factors play a critical role in regulating this protective T cell niche in the TME. For instance, IL-10 and TGF-β have been implicated in the maintenance of T_RM_ populations in tumours [222,223,224], while the induction of TCF7 gene expression (a key transcription factor that regulates T cell stemness and longevity) has also been shown to be controlled by a core transcription factor group that includes the master Th2 regulator, GATA3 [225]. Moreover, type-II inflammatory cytokines such as TSLP regulate the balance of antigen-specific memory CTLs by cooperating with IL-7 signalling [54]. Conversely, cytokines such as IL-12 and IFN-I can counteract the generation of long-lived memory T cells to favour terminal differentiation and eventual exhaustion when produced in excess [226,227,228], suggesting strong pro-inflammatory cues within the TME may drive the formation of short-lived effector CTL responses that are unfavourable in establishing anti-tumour immunity (Figure 3).

## 8. Inhibitory Receptors

### 8.1. T Cell Immunoglobulin and Mucin Domain-Containing 3 (TIM-3)

T cell immunoglobulin and mucin domain-containing 3 (TIM-3) is a type-I transmembrane protein and member of the immunoglobulin (Ig) superfamily that is upregulated on activated T cells and associated with a terminally differentiated effector state. TIM-3 has been shown to interact with several ligands including galectin-9 (Gal-9), phosphatidylserine (PtdSer), high mobility group box protein B1 (HMGB1) and carcinoembryonic antigen related cell adhesion molecule 1 (CEACAM-1) [229]. Interaction of TIM-3 with its ligand’s triggers phosphorylation of two tyrosine residues, releasing human leukocyte antigen B (HLA-B)-associated transcript 3 (BAT3) and allowing TIM-3 to exert its inhibitory function [230]. TIM-3 expression is dependent on the type-I master regulator, T-box transcription factor 21 (T-BET) [231]. Under inflammatory conditions, IL-12 and IL-27 induces TIM-3 expression and T cell dysfunction via T-BET and nuclear factor, interleukin-3 regulated (NFIL3), respectively [232,233]. In CTLs, signalling via the nuclear factor of activated T cells (NFAT) has been shown to play a role in also regulating TIM-3 expression and subsequent exhaustion [234]. It has recently been demonstrated that the interaction of TIM-3 with CEACAM1 is required for the T cell inhibitory function of TIM-3 in cancer [235]. In addition, TIM-3 is also expressed at high levels on tumour-infiltrating Tregs [236,237,238] and their presence is associated with advanced disease and the nodal metastasis in patients with NSCLC [236]. High levels of TIM-3 expression in the TME correlate with suppression of T cell responses and T cell dysfunction in cancer [239,240]. In line with this, a recent meta-analysis demonstrates that TIM-3 expression is significantly associated with worse overall survival in patients with solid cancer [241]. Consistent with a role in the negative regulation of anti-tumour immunity, there is extensive pre-clinical data demonstrating the therapeutic benefit of blocking TIM-3 signalling, mostly in conjunction with PD-1 blockade [242]. These encouraging pre-clinical results have led to development of TIM-3 blocking antibodies for clinical use, with several studies reporting early clinical findings and strong safety profiles when used in combination with αPD-1/PD-L1 or decitabine [243,244,245].

### 8.2. Lymphocyte Activation Gene-3 (LAG-3)

Lymphocyte activation gene-3 (LAG-3; CD223) is member of the Ig superfamily of receptors with structural similarities to CD4. LAG-3 conventionally binds to MHC II. however, there is also evidence of its interaction with galectin-3 (Gal-3), LSECtin, and fibrinogen like protein 1 (FGL1) [246]. LAG-3 acts as a TCR co-receptor that is upregulated following antigen exposure [247]. In line with this, IL-12 a potent inducer of IFNγ, is known to upregulate LAG-3 expression on activated human T cells along with IL-2 and IL-7 [248]. In the TME, chronic antigen stimulation and inflammation maintains LAG-3 expression on T cells and where it is commonly co-expressed with other immune checkpoints such as PD-1 and Tim-3 [249,250]. In addition, LAG-3 is constitutively expressed on suppressive Tregs [251] where it interacts with MHC II on DCs inhibiting proliferation and maturation [252]. LAG-3^+^ Tregs in the TME secrete high levels of immunosuppressive cytokines, IL10 and TGFβ, which act to dampen the anti-tumour immune response and magnify Treg activity [252]. In murine studies, blockade of LAG-3 alone or in combination with anti-PD-1 has been shown to enhance the anti-tumour CTL response and inhibit tumour growth [253,254,255]. In humans, a meta-analysis revealed that elevated LAG-3 expression is consistently associated with poor prognosis across multiple cancers [256]. Due to the fundamental role of LAG-3 plays in T cell dysfunction, early in-human clinical trials have demonstrated promising results of LAG-3 blockade in combination with αPD-1 in patients with advanced malignancies [257,258,259]. In addition to its membrane-bound form, LAG-3 can be shed from the surface by proteases, yielding soluble LAG-3 (sLAG-3). Notably, sLAG-3 has been demonstrated to elicit an immunostimulatory effect on APCs which promotes robust type-I and tumour-reactive CTL responses [260,261,262]. Indeed, high levels of sLAG-3 is associated with improved prognosis in patients with gastric cancer and correlates with serum levels of IL-12 and IFNγ [263]. Harnessing this feature of sLAG-3, clinical trials utilising recombinant sLAG-3 together with ICB have also shown encouraging results in early clinical trials [264,265], highlighting its potential role as a therapeutic adjuvant.

### 8.3. T cell Immunoreceptor with Ig and ITIM Domain (TIGIT)

T cell immunoreceptor with Ig and immunoreceptor tyrosine-based inhibition motif (ITIM) domains (TIGIT) belongs to the family of poliovirus receptor (PVR)-like proteins and is expressed on activated T cells, Tregs and NK cells [266]. TIGIT, which normally binds CD155 and CD112 on DCs and tumour cells, exerts its immunosuppressive function through numerous mechanisms, such as its direct binding to tumour expressing CD155 to trigger T/NK cell inhibition, outcompeting its co-stimulatory counterpart, CD226 on the T/NK cell surface and by indirect means including the activation of immunosuppressive DCs and Tregs following CD155-CD226 recognition [267]. Indeed, overexpression of TIGIT has been associated with poor prognosis in numerous cancers including bladder, gastric, lung adenocarcinoma and HCC [268,269,270,271]. Like other inhibitory receptors, TIGIT expression is a hallmark of T cell exhaustion [272]. Kurtulus et al. highlighted that elevated TIGIT demarcates a highly dysfunctional CTL subset in tumours, while demonstrating that TIGIT signalling orchestrates the expansion of Tregs that dampen local anti-tumour immunity [273]. Notably, tumour CD155 (the binding partner for TIGIT) expression has been linked with resistance to αPD-1 therapy in patients with metastatic melanoma [274], while expression of CD155 on cancer cells ablates CTL activation via CD226 degradation [275]. Overcoming this, dual blockade of PD-1/TIGIT could successfully recapitulate anti-tumour CTL and NK cell responses in pre-clinical mouse models [276,277,278]. Collectively, encouraging pre-clinical data has prompted the commencement of numerous clinical trials utilising αTIM-3, αLAG-3 and αTIGIT as a new class of checkpoint inhibitors for patients with treatment-refractory cancers (Table 1).

### 8.4. Cytotoxic T Lymphocyte-Associated Antigen 4 (CTLA-4)

CTLA-4 (CD152) is a member of the Ig superfamily and is highly expressed on activated T cells and constitutively expressed on Tregs [279]. The primary function of CTLA-4 is to outcompete and block CD28 co-stimulation by binding the B7 molecules on APCs (CD80/CD86) to elicit downstream T cell inhibition [280]. Cell-extrinsic mechanisms also come into play by stripping B7 molecules from APCs following CTLA-4 engagement via trans-endocytosis [281], and reverse CTLA-4-B7 signalling that activates immunosuppressive IDO activity in DCs [282]. Furthermore, CTLA-4 plays a pivotal role in maintenance of Tregs and their immunosuppressive activity [283]. In humans, the correlation between high levels of CTLA-4 expression and poor outcome is well established in several types of cancer [284]. In pre-clinical studies, blockade of CTLA-4 led to rejection of lymphoma, colorectal, renal and fibrosarcoma cancer cell lines in mice [285] and activation of human tumour-specific CTLs [286]. These studies led to the development of ipilimumab, a fully human αCTLA-4 IgG1 monoclonal antibody (mAb), that demonstrated improved survival outcomes in a key phase 3 study of patients with previously treated metastatic melanoma [287]. This pivotal trial led to Ipilimumab being the first immune checkpoint blockade therapy approved by the Food and Drug Administration (FDA) in 2011 for patients with advanced melanoma, a disease stage for which there was no previous standard of care therapy that prolonged survival. Since then, αCTLA-4 blockade has been examined extensively in pre-clinical studies and clinical trials as a single agent and in combination with chemotherapy [288,289,290], radiotherapy [291,292,293], cancer vaccination [294], and other immunotherapies [295,296,297]. However, it is the combination of αPD-1/αCTLA-4 blockade that has demonstrated the greatest therapeutic efficacy in the clinic, with ipilimumab (αCTLA-4) and nivolumab (αPD-1) approved for treatment of patients with advanced solid tumours (Table 2).

### 8.5. Programmed Cell Death Protein 1 (PD-1)

PD-1 (CD279) is another membrane-bound co-inhibitory receptor with Ig-like domains. However, unlike CTLA-4, PD-1 expression is more broadly expressed across hematopoietic and non-hematopoietic cells. PD-1 binds its cognate ligands, PD-L1 and PD-L2 which are membrane proteins also found on numerous cell types including APCs, endothelial cells, cancer cells, mast cells and lymphocytes [298]. In T cells, PD-1 engagement by PD-L1/PD-L2 triggers several immunosuppressive mechanisms by interfering with both downstream TCR and CD28 signalling, while directly inducing the expression of regulatory transcription factors such as basic leucine zipper transcriptional factor ATF-like (BATF) [299]. In the TME, PD-1 is highly expressed on tumour-infiltrating lymphocytes, where it is commonly associated with a population of dysfunctional or “exhausted” T cells that co-express multiple immune checkpoint molecules and display a unique epigenetic landscape compared with effector and memory T cells [300]. The expression of PD-L1 on tumour cells has been studied extensively as a mechanism of subverting T cell immunity, which has been reported in a variety of solid cancers [301] and often associated with poor overall survival [302]. The success of targeting the PD-1/PD-L1 signalling axis in pre-clinical studies lead to the prompt development mAbs against this pathway in the clinic. Pembrolizumab (Keytruda; Merck) is a humanized IgG4 αPD-1 monoclonal antibody that was the first PD-1 targeting immunotherapy to receive FDA approval in 2014 for treatment of patient with advanced melanoma [303,304]. Since then, PD-1/PD-L1 inhibitors, commonly paired with CTLA-4 blockade (ipilimumab) have been used extensively across many advanced solid cancers with modest clinical efficacy (Table 2). Despite its success, resistance to ICB is routinely observed among cancer patients, where lower levels of mutation burden, and reduced intra-tumoural PD-1/PD-L1 and MHC-I expression are common drivers of ICB resistance [305]. In addition, the use of αPD-1/PD-L1 upregulates alternative checkpoint molecules such as TIM-3 which may also be targetable in combination therapies [306]. Interestingly, chronic IFN signalling in the TME leads to the upregulation of tumour-derived inhibitory molecules such as PD-L1 which act to resist ICB and promote CTL dysfunction [307]. While IFNs have proven to be a critical axis in eliciting anti-tumour immunity, it is conceivable that sustained IFN signalling confers several adaptive resistance programs to ICB due its ability to exert strong selective pressures.

## 9. Conclusions

**Figure 4:**
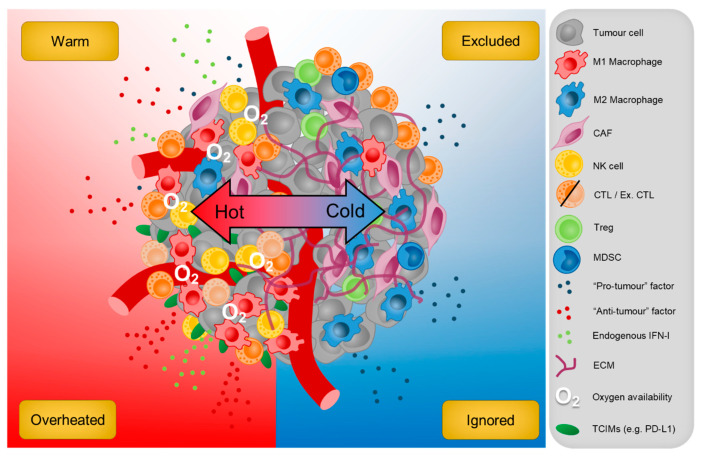
The cellular composition of ‘hot’ and ‘cold’ tumours. Anti-tumour immunity in the TME depends on the presence of CTLs that are activated by endogenous IFN-I and other pro-inflammatory stimuli produced by neighbouring cells. However, these can be subverted by an imbalance of anti-inflammatory factors that also reduces their trafficking to the tumour site. Conversely, too much inflammation may impair the cytolytic activities of CTLs, triggering immune escape. This intense inflammation mediated by conventional anti-tumour factors such as IFN-Is also upregulate T cell inhibitory molecules (TCIMs) on tumour cells that drive the adaptive resistance to immunotherapy.

Solid tumours display remarkable heterogeneity both within and across various cancer types, which reflects the diversity of response rates to immunotherapy between patients [308]. Current advances in precision medicine have enabled the stratification of those that are more likely to benefit from immunotherapy. For instance, the quantitative measurement of CD3^+^ T cells within the tumour core and invasive margin has been useful in predicting clinical responses to treatment in patients with colorectal cancer [309], while other work has shown that an absence of intra-tumoural PD-L1 expression is more likely to blunt the therapeutic effects of αPD-1 ICB [310]. Another well-recognised predictor of treatment response also includes tumour mutational burden (TMB), where cancer types that exhibit higher rates of TMB, such as melanoma and cutaneous squamous cell tend to benefit the most from ICB [308]. These metrics do have their limitations and still cannot account for the vast majority of individuals that fail to respond to therapy. However, with the advent of next generation sequencing (NGS), new insights can now be garnered by unveiling the complex intercellular networks at exquisite single-cell resolution in the TME. Although it is indisputable that some molecular pathways clearly subvert tumour immunosurveillance (i.e., hypoxia and inhibitory receptor expression); many have both pro- and anti-tumour activities. It is therefore intuitive that a ‘hot’ and ‘cold’ TME is an overly simplistic binary representation of the local immune contexture and that additional tumour subtypes across this spectrum need to be investigated further (Figure 4). Our review highlights the necessity of balancing pro- and anti-inflammation in the TME to mobilise the host immune system against cancer and establish long-term anti-tumour immunity. With the use of modern molecular profiling techniques, the identification of patients with varying degrees of tumour inflammation, including those that are potentially ‘overheated’ will lead to the improvement of more personalised therapeutics that maximise clinical responses for patients with advanced solid tumours.

## Figures and Tables

**Table 1:** Clinical trials underway using therapeutics against TIM-3, LAG-3 and TIGIT (clinicaltrials.gov).

Product	Description	Clinical Stage	Cancer(s)	Trial No.
**A. TIM-3**				
Sym023 (Symphogen)	IgG1 mAb	1	Advanced solid cancer or lymphoma	NCT03489343 NCT03311412
TSR-022 (Tesaro)	Humanised IgG4 mAb	2	HCC, melanoma, advanced solid cancers	NCT03680508 NCT02817633 NCT03307785
LY3321367 (Eli Lilly and Co)	IgG1k, Fc silent	1	Melanoma, MSI-H and advanced solid cancer	NCT03099109 NCT02791334
MBG453 (Novartis)	Humanised IgG4 mAb	2	AML, CMML-2, MDS, GBM	NCT04150029 NCT04266301 NCT03946670 NCT03066648 NCT03940352 NCT02608268 NCT03961971
BGB-A425 (BeiGene)	IgG1, variant, Fc silent	1/2	Advanced/ Metastatic solid cancer	NCT03744468
ICAGN02390 (Incyte)	IgG1k, N297A, Fc silent	1	Advanced solid cancer, melanoma	NCT03652077 NCT04370704
BMS-986258 (Bristol-Myers Squibb)	IgG1, Fc silent	1/2	Advanced solid cancer	NCT03446040
RO7121661 (Hoffman-La Roche)	PD-1/TIM-3 bispecific Ab	1	Melanoma, NSCLC, SCLC, ESCC, urothelial cancer	NCT03708328 NCT03869190
**B. LAG-3**				
IMP321 * (Immunopet)	LAG-3-Ig fusion protein	1/2	Breast cancer, advanced solid cancers, NSCLC, HNSCC	NCT02614833 NCT03252938 NCT03625323
Relatlimab ^#^ (Bristol-Myers Squibb)	IgG4 mAb	2	Uveal melanoma, CRC, sarcoma, melanoma	NCT04552223 NCT03642067 NCT04095208 NCT03470922
LAG525 (Novartis)	IgG4 mAb	2	Breast cancer, advanced solid cancer, melanoma	NCT03499899 NCT02460224 NCT03742349 NCT03484923
MK-4280 (Merck)	Humanised IgG4	1/2	HL, NHL, BCL, NSCLC, RCC, advanced solid cancer	NCT03598608 NCT02720068 NCT03516981 NCT04626479 NCT04626518
REGN3767 (Regeneron)	IgG4 mAb	1	BC, advanced cancers	NCT03005782 NCT01042379
TSR-033 (Tesaro)	Humanised IgG4 mAb	1	Advanced solid cancers	NCT03250832 NCT02817633
Sym022 (Symphogen)	Fc inert mAb	1	Advanced solid cancer, lymphoma	NCT04641871 NCT03311412
INCAGN02385 (InCyte)	Fc-engineered IgG1k	1/2	Melanoma	NCT04370704
MGD013 (MacroGenics)	Humanised LAG3-PD-1 bispecific Ab	1/2	GC, HCC, advanced solid cancers	NCT04212221 NCT03219268 NCT04178460
FS118 (F-star)	Humanised LAG3-PD-L1 bispecific Ab	1	Advanced solid cancer	NCT03440437
**C. TIGIT**				
BMS-986207 (Bristol-Myers Squibb)	IgG1 mAb, FcγR null	1/2	EC, OC, Myeloma, advanced solid cancer,	NCT04570839 NCT02913313 NCT04150965
IBI939 (Innovent)	IgG1 mAb	1	Advanced cancer	NCT04353830
BGB-A1217 (BeiGene)	Humanized IgG1 mAb	1	Metastatic solid tumours	NCT04047862
Tiragolumab ^†^ (Genentech)	IgG1 mAb	1–3	NSCLC, SCLC, ESCC, BC	NCT03563716 NCT04300647 NCT04543617 NCT04584112
Etigilmab (Mereo BioPharma)	Humanised IgG1 mAb	1	Advanced solid cancer	NCT03119428
Vibostolimab (Merck)	Humanised IgG1 mAb	1/2	GC, NSCLC, PC, melanoma,	NCT02964013 NCT02861573 NCT04305054 NCT04303169 NCT04165070 NCT04305041
Domvanalimab (Arcus Biosciences)	Humanised IgG1 mAb	1	Advanced solid cancer, NSCLC	NCT03628677 NCT04262856
ASP8374 (Potenza)	IgG4 mAb	1	Advanced solid cancer	NCT03260322 NCT03945253

^#^ 28 studies recruiting/active, not recruiting, 4 not yet recruiting, 1 withdrawn; * 3 studies recruiting/active, not recruiting, 6 completed, 1 not yet recruiting; ^†^ 13 studies recruiting, 1 active, not recruiting, 1 not yet recruiting. Abbreviations: HCC: heptocellular carcinoma; MSI-H: microsatellite instability high; AML: acute myeloid leukemia; CMML: chronic myelomonocytic leukemia; MDS: myelodysplastic syndrome; GBM: glioblastoma multiforme; NSCLC: non-small-cell lung cancer; SCLC: small-cell lung cancer; ESCC: esophageal squamous cell carcinoma; HNSCC: head and neck squamous cell carcinoma; CRC: colorectal cancer; HL: Hodgkin’s lymphoma, NHL: non-Hodgkin’s lymphoma; BCL: B cell lymphoma; RCC: renal cell carcinoma; BC: breast cancer; GC: gastric cancer; EC: endometrial cancer; OC: ovarian cancer; PC: prostate cancer.

**Table 2:** Clinical applications of αCTLA-4/αPD-1 drugs combinations and the seminal studies that lead to their FDA approval (clinicaltrials.gov and fda.gov).

Product	Combination(s)	Cancer Type(s)	Seminal Study
**A. CTLA-4**			
Ipilimumab	Nil	Melanoma	NCT00094653
(Bristol-Myers Squibb)	Surgery	Melanoma	NCT00636168
	Nivolumab	Melanoma	NCT01844505
	Nivolumab	RCC	NCT02231749
	Nivolumab	MSI-H/dMMR CRC	NCT02060188
	Nivolumab	HCC	NCT01658878
	Nivolumab and limited Chemotherapy	NSCLC	NCT03215706
	Nivolumab	Malignant pleural mesothelioma	NCT02899299
**B. PD-1**			
Cemiplimab (Regeneron)	Nil	Cutaneous SCC	NCT02760498
Pembrolizumab	Nil	Melanoma	NCT01866319
(Merck)	Surgery	Melanoma	NCT02362594
	Nil	NSCLC	NCT01295827
			NCT02220894
	Doublet platinum-based Chemotherapy	NSCLC	NCT02039674
	Carboplatin and paclitaxel	NSCLC	NCT02775435
	Axitinib	RCC	NCT02853331
	Nil	MSI-H/dMMR CRC	NCT02563002
	Nil	SCLC	NCT02054806
			NCT02628067
	Platinum and FU	HNSCC	NCT01848834
	Nil	HNSCC	NCT02358031
	Nil	Gastric cancer	NCT02335411
	Nil	ESCC	NCT02564263
	Nil	HCC	NCT02702414
	Nil	MCC	NCT02267603
	Lenvatinib	Endometrial cancer	NCT02501096
	Nil	cSCC	NCT03284424
	Paclitaxel or gemcitabine and carboplatin	TNBC	NCT02819518
	Nil	Urothelial cancer	NCT02335424
			NCT02625961
	Nil	PMBCL	NCT02576990
	Nil	Classical HL	NCT02453594
			NCT02684292
Nivolumab	Nil	NSCLC	NCT01642004
(Bristol-Myers Squibb)	Nil	RCC	NCT01668784
	Nil	Classical HL	NCT02181738
			NCT01592370
	Nil	HNSCC	NCT02105636
	Nil	Urothelial Carcinoma	NCT02387996
	Nil	MSI-H/dMMR CRC	NCT02060188
	Nil	HCC	NCT01658878
	Surgery	Melanoma	NCT02388906
	Ipilimumab	SCLC	NCT01928394
	Nil	ESCC	NCT02569242

NSCLC: non-small-cell lung cancer; RCC: renal cell carcinoma; MSI-H: microsatellite instability high; dMMR: mismatch repair deficient; CRC: colorectal cancer; SCLC: small-cell lung cancer; HNSCC: head and neck squamous cell carcinoma; ESCC: esophageal squamous cell carcinoma; MCC: Merkel cell carcinoma; cSSC: cutaneous squamous cell carcinoma; TNBC: triple-negative breast cancer; PBMCL: Primary mediastinal large B-cell lymphoma; HL: Hodgkin’s lymphoma: TMB-H: tumour mutational burden high.

## Data Availability

Not applicable.
